# Changes in cellular microRNA expression induced by porcine circovirus type 2-encoded proteins

**DOI:** 10.1186/s13567-015-0172-5

**Published:** 2015-04-10

**Authors:** Jae-Sang Hong, Nam-Hoon Kim, Chang-Yong Choi, Jun-Seong Lee, Dokyun Na, Taehoon Chun, Young Sik Lee

**Affiliations:** College of Life Sciences and Biotechnology, Korea University, Seoul, 136-713 Korea; School of Integrative Engineering, Chung-Ang University, Seoul, 156-756 Korea; Present address: Institut de Recherches Cliniques de Montréal, Montréal, Québec, H2W1R7 Canada

## Abstract

Porcine circovirus type 2 (PCV2) is the primary causative agent of postweaning multisystemic wasting syndrome, which leads to serious economic losses in the pig industry worldwide. While the molecular basis of PCV2 replication and pathogenicity remains elusive, it is increasingly apparent that the microRNA (miRNA) pathway plays a key role in controlling virus-host interactions, in addition to a wide range of cellular processes. Here, we employed Solexa deep sequencing technology to determine which cellular miRNAs were differentially regulated after expression of each of three PCV2-encoded open reading frames (ORFs) in porcine kidney epithelial (PK15) cells. We identified 51 ORF1-regulated miRNAs, 74 ORF2-regulated miRNAs, and 32 ORF3-regulated miRNAs that differed in abundance compared to the control. Gene ontology analysis of the putative targets of these miRNAs identified transcriptional regulation as the most significantly enriched biological process, while KEGG pathway analysis revealed significant enrichment for several pathways including MAPK signaling, which is activated during PCV2 infection. Among the potential target genes of ORF-regulated miRNAs, two genes encoding proteins that are known to interact with PCV2-encoded proteins, *zinc finger protein 265* (*ZNF265*) and *regulator of G protein signaling 16* (*RGS16*), were selected for further analysis. We provide evidence that *ZNF265* and *RGS16* are direct targets of miR-139-5p and let-7e, respectively, which are both down-regulated by ORF2. Our data will initiate further studies to elucidate the roles of ORF-regulated cellular miRNAs in PCV2-host interactions.

**Electronic supplementary material:**

The online version of this article (doi:10.1186/s13567-015-0172-5) contains supplementary material, which is available to authorized users.

## Introduction

Porcine circoviruses (PCVs) are small, non-enveloped viruses with a circular single-stranded DNA genome of approximately 1.7 kb [1]. Two types of PCV have been described. The original virus, designated PCV type 1 (PCV1), is non-pathogenic to pigs [2], while a variant strain of PCV, designated PCV type 2 (PCV2), is the principal etiological agent of postweaning multisystemic wasting syndrome (PMWS), a multifactorial disease in swine that leads to severe losses in pig production worldwide [3]. Prominent PMWS symptoms include severe progressive weight loss, dyspnea, tachypnea, anemia, diarrhea, and lymphocyte depletion in pigs between 5 and 15 weeks of age [4,5]. PCV2 infections are also associated with other porcine diseases, such as porcine dermatitis and nephropathy syndrome (PDNS) and porcine respiratory disease complex (PRDC) [6]. Despite the severe consequences of PCV2 infection, the mechanisms underlying replication and pathogenesis of PCV2 have remained elusive.

Replication of PCV2 involves the generation of a double-stranded DNA intermediate, which encodes three major open reading frames (ORFs) on both the viral (ORF1) and the complementary (ORF2 and ORF3) strands [7]. ORF1 encodes the two replication-associated proteins (Rep and Rep’) via alternative splicing, which are both necessary for viral DNA replication [8]. ORF2 codes for the immunogenic capsid (Cap) protein [9]. ORF3 is expressed in the antisense direction of ORF1 and encodes a protein that is not essential for viral replication but contributes to caspase-dependent apoptosis of host cells and modulation of virulence [10,11].

Since PCV2 has a highly limited coding capacity due to its small genome size, replication and pathogenesis of PCV2 are largely dependent on host factors. PCV2-encoded proteins were found to interact with several cellular proteins involved in transcriptional regulation as well as components of signaling pathways [12-14], implicating modulation of host transcriptional regulatory networks in augmenting the replication potential of PCV2. For example, the transcriptional regulator c-Myc was found to interact with the Rep protein of PCV2 [12]. Importantly, c-Myc modulates the expression of several microRNAs (miRNAs), which are key regulators of gene expression [15-17]. Based on these findings, it is plausible that the viral proteins expressed during PCV2 infection lead to differential regulation of cellular miRNAs.

miRNAs are an abundant class of ~22-nucleotide (nt) non-coding RNAs that act as key post-transcriptional regulators of gene expression in metazoans [18], and affect almost every cellular process, from development to oncogenesis [19]. Mammalian organisms express hundreds of miRNAs [20]. Canonical miRNAs are transcribed by RNA polymerase II as long primary transcripts (pri-miRNAs), which are processed in the nucleus into ~70-nt precursor miRNAs (pre-miRNAs) with hairpin structures by the RNase III enzyme Drosha [21,22]. The pre-miRNA is exported to the cytoplasm where another RNase III enzyme named Dicer further processes it into a miRNA duplex. Each strand of this duplex originates from the 5′ and 3′ arms of a stem region in the pre-miRNA hairpin and is denoted with a -5p (from the 5′ arm) or -3p (from the 3′ arm) suffix [23]. One strand of the miRNA duplex, representing a mature miRNA, is incorporated into the RNA-induced silencing complex (RISC) to direct translational repression and/or destabilization of target mRNAs primarily by binding to their 3′ untranslated region (3′ UTR) [24]. In animals, positions 2 to 7 from the 5′ end of the miRNA, referred to as the ‘seed’ region, are the major determinants for RISC binding to its partially complementary targets [20]. As individual miRNAs can regulate multiple genes [25], alteration of miRNA expression has been associated with numerous human diseases including cancer [26].

Increasing evidence indicates that viruses modulate cellular miRNA expression profiles upon host infection [27-29]. Following viral infections, altered expression of cellular miRNAs can facilitate and/or restrict viral replication by deregulating their target genes involved in cell proliferation, survival, and antiviral defense pathways. For example, differential expression of cellular miRNAs induced by hepatitis C virus and human immunodeficiency virus affects viral replication and pathogenesis [28]. Nevertheless, PCV2 has previously not been shown to deregulate cellular miRNA expression upon infection.

PCV2-encoded proteins are major interfaces through which the virus interacts with host cells and modulates their activity to establish infection. In this study, we characterized cellular miRNAs that are either up-regulated or down-regulated in porcine kidney epithelial (PK15) cells by each of three PCV2-encoded ORF proteins using Solexa deep sequencing technology. We also performed gene ontology (GO) and KEGG pathway analyses to identify key cellular processes and pathways associated with the putative target genes of ORF-regulated miRNAs. Moreover, we further analyzed two target genes of ORF-regulated miRNAs that encode proteins known to interact with PCV2-encoded proteins. Our results can be used as a platform to study the functions of cellular miRNAs associated with PCV2 replication and pathogenesis.

## Materials and methods

### Cell culture and generation of stable cell lines

PK15 cells were maintained at 37 °C in Dulbecco’s Modified Eagle Medium (DMEM; Hyclone) with 10% fetal bovine serum (FBS; Hyclone) in an atmosphere of 5% CO_2_. Genomic DNA was extracted from a PCV2 strain [GenBank accession no. FR823451.1], isolated from the spleen of a pig obtained from a commercial farm in South Korea. To generate PK15 cell lines stably expressing each PCV2 ORF, individual full-length ORFs were amplified from the PCV2 genomic DNA by PCR using ORF1, ORF2, or ORF3 primer pairs (Additional file 1) containing *Xho*I and *Not*I restriction sites. After digestion with *Xho*I and *Not*I, each of the resulting PCR products was cloned into the pGEM-T Easy vector (Promega), and the nucleotide sequence was verified by DNA sequencing. Each ORF was then subcloned into the *Xho*I and *Not*I sites downstream of the cytomegalovirus (CMV) promoter in the pLNCX2 retroviral vector (Clontech). To generate retroviruses, 293 GPG packaging cells were transfected with either the empty vector or individual recombinant vectors using Lipofectamine Plus (Invitrogen), according to the manufacturer’s protocol. Three days after transfection, the supernatant of the transfected cells containing retroviruses was collected and used to infect PK15 cells in the presence of 1 μg/mL polybrene (Sigma). Four hours after infection, the viral supernatant was replaced with DMEM containing 10% FBS. The retroviral infection procedure of PK15 cells was performed three times at 24 h intervals. After the third infection, the cells were selected with 1.5 mg/mL neomycin to establish stable cell lines.

### Reverse transcription-polymerase chain reaction (RT-PCR)

Total RNA was extracted from PCV2 ORF-expressing and control PK15 cells using Trizol reagent (Invitrogen) according to the manufacturer’s protocol. One microgram of total RNA from each sample was treated with RNase-free DNase I (Invitrogen), and reverse-transcribed to cDNAs using random primers (Promega) and M-MLV reverse transcriptase (Invitrogen) according to the manufacturer’s instructions. The ORF1 cDNA was PCR amplified using either the ORF1/3-RT-F and ORF1-RT-R primer pair or the ORF1/3-RT-F and ORF1/3-RT-R primer pair. The ORF2 cDNA was amplified with the ORF2-RT-F and ORF2-RT-R primer pair, and the ORF3 cDNA was obtained using the ORF1/3-RT-F and ORF1/3-RT-R primer pair. GAPDH (glyceraldehyde-3-phosphate dehydrogenase) cDNA was amplified by PCR using the GAPDH primer pair, which served as an internal control. The sequences of all primers are listed in Additional file 1.

### Small RNA cDNA library construction and Solexa sequencing

Total RNA was isolated from each sample using the miRNeasy mini Kit (Qiagen) and then enriched for small RNAs less than ~200 nt using an RNeasy MinElute Cleanup kit (Qiagen), according to the manufacturer’s instructions. The small RNAs were measured for their integrity and quantity on an Experion system (Bio-Rad) and then used as input material to construct a cDNA library using a TruSeq Small RNA Sample Preparation kit (Illumina) following the manufacturer’s protocol. Briefly, one microgram of small RNAs was sequentially ligated to 3′ and 5′ RNA adaptors. The doubly ligated RNA products were purified and reverse-transcribed to cDNAs, followed by 11 cycles of PCR using a pair of common and index primers. The resulting libraries were gel-purified and quantified using picoGreen and qPCR [30], and their size and quality were assessed with Experion in combination with Bioanalyzer 2100 (Agilent). Each library (8 pM) was used for cluster generation with a TruSeq SR cluster kit v2 (Illumina) on an Illumina cBot, followed by sequencing on an Illumina Genome Analyzer IIx. Solexa sequencing data were submitted to the GEO database (accession number GSE60206).

### Computational processing of Solexa sequencing data

Raw sequencing reads from each library were subjected to the small RNA data-processing pipeline of the Beijing Genomics Institute (BGI, China). After eliminating all low-quality sequences, the reads between 18 and 36 nts were retrieved and trimmed of the adaptor sequences to produce “clean reads”. The filtered datasets were analyzed for small RNA length distribution and then aligned to the porcine genome (Sscrofa10.2) using the SOAP program (version 2.20) [31]. Next, all clean reads were screened against public databases for annotation. To avoid redundant annotation of the reads, bioinformatic analysis was performed in the following order: non-coding RNAs other than miRNAs > miRNAs > repeat-associated small RNAs > mRNAs. The Rfam and NCBI GenBank databases were used to identify sequences matching repeats, mRNAs, and non-coding RNAs (e.g., rRNA, tRNA, snRNA, and snoRNA) other than miRNAs. To identify known porcine miRNAs, the total clean reads from each library were aligned to porcine pre-miRNAs and mature miRNAs annotated in miRBase (release 20.0) [32] using BLASTN. Only reads perfectly matching pre-miRNAs, but partially matching their corresponding mature miRNAs with at least 16 nt overlap, were considered known porcine miRNA variants, termed isomiRs [33].

To identify porcine orthologs of human miRNAs and their respective isomiRs, the total clean reads from each library were compared to human pre-miRNAs and their corresponding mature miRNAs, registered in miRBase, using the standalone version of miRanalyzer [34], allowing no mismatch and a minimum 16-nt contiguous match, respectively. If a read was perfectly mapped to both a known porcine mature miRNA and a human mature miRNA (except for a 1 or 2-nt mismatch at either the 5′ or 3′ end), it was considered an identical miRNA, conserved between pigs and humans. If the remaining reads, matching both human pre-miRNAs and their mature miRNAs, perfectly mapped to the porcine genome, the genomic sequence, including flanking regions, was used to predict hairpin structures of 70–80 nt with a free energy of less than −20 kcal/mol using the mfold program (version 3.5) [35]. Any sequence that fulfilled the criteria for a potential miRNA hairpin precursor was considered a porcine ortholog of human miRNA [36].

To compare miRNA expression profiles between samples (control versus ORF-expressing PK15 cells), the abundance levels for individual miRNAs in each library were normalized by dividing each miRNA count by the total number of clean reads as described previously [37]. The normalized ORF/control ratios were log_2_ transformed to identify miRNAs with at least a two-fold change in expression. Raw miRNA read counts were also statistically analyzed for differential expression with the Fisher’s exact test (*P* < 0.05). miRNAs that satisfied these criteria were considered PCV2 ORF-regulated miRNAs and subjected to further analysis.

To identify miRNA clusters, pre-miRNA sequences were retrieved from miRBase and mapped to the porcine genome. The genome-matched sequences were used to identify clusters of individual miRNAs that were located in close proximity (<10 kb apart) on a chromosome and oriented in the same direction for transcription.

### Prediction of miRNA targets and functional enrichment analysis

Potential target genes of PCV2 ORF-regulated miRNAs were predicted using miRecords, a resource for miRNA-target interactions that integrates 11 miRNA target prediction programs including TargetScan, miRanda, and PicTar [38]. Due to the lack of porcine genes in the current version of miRecords, human orthologs of porcine miRNAs with differential expression were used to predict potential target genes, assuming that the 3′ UTRs of orthologous mRNAs between humans and pigs contain conserved miRNA-binding sites. Genes that were predicted by at least five of the target prediction programs integrated into miRecords were considered the most probable targets of the ORF-regulated miRNAs. For human miRNA targets of particular interest, the 3′ UTR sequences of orthologous mRNAs in pigs, if available, were retrieved from NCBI and analyzed to confirm the conserved miRNA-target interactions using RNAhybrid [39]. Sites complementary to porcine miRNAs with seed matches and free energies of at least −20 kcal/mol for hybridization were considered miRNA target sites. GO biological processes and KEGG pathways enriched in the predicted miRNA target gene datasets were identified with DAVID (version 6.7) [40] using the criteria that at least ten genes were involved and there was a *P* < 0.05 for each category.

### miRNA expression analysis

Splinted ligation assay was performed as described previously using total RNA (2 μg) extracted from each sample to measure mature miRNA levels [41-43]. Equal amounts of input RNA for reactions were further verified by visualizing 5.8S RNA as an internal control with ethidium bromide after electrophoresis of total RNA on denaturing polyacrylamide gels. The sequences of miRNA-specific bridge oligonucleotides used for the splinted ligation assay are listed in Additional file 2. Reaction products were resolved on 12% polyacrylamide gels containing 7 M urea, visualized on a BAS-2500 Phosphorimager (Fujifilm), and quantified using MultiGauge software (Fujifilm).

### Western blotting

Protein extracts were prepared in passive lysis buffer (50 mM Tris–HCl (pH 7.4), 150 mM NaCl, 0.5% NP-40, and protease inhibitor (Roche)) from PCV2 ORF-expressing and control PK15 cells. Western blot analysis and quantification were performed as described previously [44]. The primary antibodies used were anti-RGS16 (Santa Cruz) and anti-α-Tubulin (Santa Cruz), which was used as a loading control.

### Luciferase reporter assays

To construct a plasmid expressing miR-139-5p or let-7e, a fragment containing the corresponding miRNA precursor was amplified from genomic DNA of PK15 cells by PCR with the miR-139 or let-7e primer pair (Additional file 1) and cloned into the pCI-neo vector (Promega). To generate luciferase reporter constructs, a fragment of either the *ZNF265* 3′ UTR [GenBank accession no. NM_001044582.1] or *RGS16* 3′ UTR [GenBank accession no. AK399836.1] was obtained from PK15 cell-derived cDNAs by PCR with the ZNF265 WT or RGS16 primer pair (Additional file 1), and cloned downstream of the *Renilla* luciferase-coding sequence in the psiCHECK-2 vector (Promega), which also expresses firefly luciferase for normalization of the *Renilla*-luciferase activity between samples. Site-directed mutagenesis of a miR-139-5p target site in the *ZNF265* 3′ UTR was performed using PfuTurbo DNA polymerase (Stratagene) and the ZNF265 mutant primer pair listed in Additional file 1. For luciferase assays, control or ORF2-expressing PK15 cells were co-transfected in 12-well plates with the luciferase-3′ UTR reporter plasmid (wild type or mutant) and the pCI-neo vector containing the corresponding miRNA precursor or vector only using Lipofectamine 2000 (Invitrogen) according to the manufacturer’s instructions. Two days after transfection, *Renilla*- and firefly-luciferase activities were measured using the Dual-Luciferase Reporter Assay System (Promega), according to the manufacturer’s protocol.

## Results

### Solexa sequencing of small RNA cDNA libraries and data analysis

**Figure 1:**
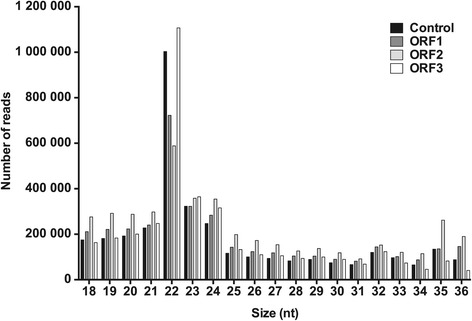
**Size distribution of small RNA sequences.** The clean reads ranging from 18-nt to 36-nt in size were selected from each small RNA cDNA library after deep sequencing, as described in Materials and Methods. The majority of them were 22 nt, which is consistent with the typical size of miRNAs.

Since other viruses use cellular miRNA to modulate host-cell infection, we hypothesized that PCV2 also alters the miRNA expression profile of host cells during infection. To address this possibility, we expressed individual PCV2-encoded ORFs (ORF1, ORF2, and ORF3) in PK15 cells, which have been widely used to propagate PCV2 and study its replication in vitro [45,46]. This method was chosen instead of infecting these cells with PCV2 because of the low infectivity of the virus strain used in this study (data not shown). In addition, this approach might enable the analysis of the potential effect of each ORF protein on cellular miRNA expression during the course of PCV2 infection. We generated PK15 cell lines stably expressing each of three PCV2 ORFs (Additional file 3). Small RNA cDNA libraries were prepared from these cell lines as well as the parental cell line as a control, and then sequenced using a Solexa platform. Sequencing of each library yielded between 4 565 856 and 5 887 207 raw reads. After excluding low-quality reads and adaptor sequences, the remaining sequences that were 18–36 nt in length were selected as “clean reads” for further analysis. These reads accounted for an average of 75.55% of the raw reads obtained from each library. The size distribution of the clean reads was similar in all the libraries, with the majority being 22 nt, which is typical of miRNAs (Figure 1). All of the clean reads were then annotated by searching against current public databases. In our libraries, an average of 27.93% of the reads for each library were miRNAs (see below). An average of 33.89% of the total clean reads represented degradation products of mRNA and other non-coding RNAs, and an average of 38.18% remained unclassified. The details of the sequencing results of the four libraries, including read counts and relative proportion for each category, are shown in Table 1.

**Table 1:** **Sequencing results of small RNA cDNA libraries**

**Category**	**Control**		**ORF1**		**ORF2**		**ORF3**	
	**Count**	**%**	**Count**	**%**	**Count**	**%**	**Count**	**%**
**Raw reads**	**4 654 382**	**100.00**	**5 014 372**	**100.00**	**5 887 207**	**100.00**	**4 565 856**	**100.00**
Low-quality reads	42 632	0.92	45 864	0.91	57 174	0.97	40 451	0.89
High-quality reads	4 611 750	99.08	4 968 508	99.09	5 830 033	99.03	4 525 405	99.11
**High-quality reads**	**4 611 750**	**100.00**	**4 968 508**	**100.00**	**5 830 033**	**100.00**	**4 525 405**	**100.00**
Clean reads (≥18 nucleotides)	3 476 739	75.39	3 604 325	72.54	4 293 063	73.64	3 648 407	80.62
**Clean reads**	**3 476 739**	**100.00**	**3 604 325**	**100.00**	**4 293 063**	**100.00**	**3 648 407**	**100.00**
miRNA	1 205 500	34.67	861 472	23.90	623 299	14.52	1 409 182	38.62
rRNA	698 265	20.08	775 446	21.51	1 568 539	36.54	808 436	22.16
scRNA	4100	0.12	3292	0.09	2835	0.07	2541	0.07
snRNA	15 446	0.44	12 003	0.33	19 892	0.46	12 971	0.36
snoRNA	16 659	0.48	10 548	0.29	11 925	0.28	11 051	0.30
srpRNA	332	0.01	509	0.01	1428	0.03	591	0.02
tRNA	186 300	5.36	289 446	8.03	283 870	6.61	192 332	5.27
mRNA	44 231	1.27	48 588	1.35	68 330	1.59	52 302	1.43
Repeat-associated small RNA	8168	0.23	8876	0.25	10 357	0.24	9058	0.25
Unannotated^a^	1 297 738	37.33	1 594 145	44.23	1 702 588	39.66	1 149 943	31.52

^a^Sequences do not match any known RNA species.

### Identification of porcine miRNAs

**Figure 2:**
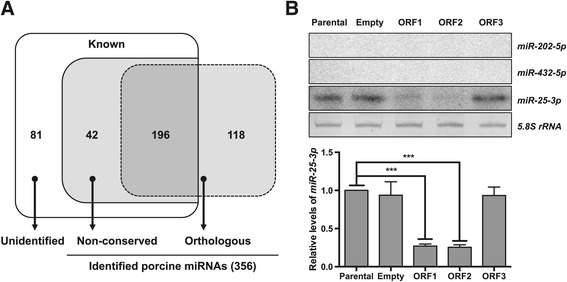
**Identification of known and orthologous miRNAs in the pig. A**. Overlap of known porcine miRNAs and the orthologs of human miRNAs identified in this study. The total number of the identified miRNAs is indicated in parenthesis. Known, porcine miRNAs annotated in miRBase; Unidentified, known porcine miRNAs not identified in this study; Non-conserved, miRNAs that are not conserved between humans and pigs; Orthologous, miRNAs conserved between humans and pigs (indicated by a dot-border rectangle). **B**. Splinted ligation analysis (top) and quantification (bottom) of expression levels of the porcine ortholog of human miR-25-3p. A representative image from three independent experiments is shown. Orthologous but unidentified porcine miRNAs, miR-202-5p and miR-432-5p, served as negative controls, and the 5.8S rRNA was used as a loading control. Consistent with Solexa sequencing results, miR-25-3p was significantly down-regulated in ORF1- and ORF2-expressing PK15 cells but not in ORF3-expressing cells, compared with parental and empty vector-harboring cells. For quantification, the level of miR-25-3p in parental cells was set to 1. All error bars indicate mean ± standard error of the mean (SEM). ***, *P* < 0.001 by one-way analysis of variance (ANOVA) with Dunnett’s multiple-comparison test.

We first identified known porcine miRNAs by searching all of the clean reads against the primary repository for miRNA sequences and annotations, miRBase (release 20.0). We observed different but overlapping sets of known porcine miRNAs across the libraries. The miRNAs varied significantly between the libraries in their relative abundance, as measured by the frequency of read counts in each library (Additional file 4), suggesting potential changes in cellular miRNA levels induced by individual PCV2 ORFs. Amongst the 319 distinct porcine miRNAs annotated in miRBase, 81 were not detected in any libraries, presumably due to very low or no expression in PK15 cells. The remaining 238 miRNAs were, however, observed in at least one of the libraries, representing 74.61% of all known porcine miRNAs (Figure 2A). Amongst these, 210 miRNAs were detected in the control library, 215 in the ORF1 library, 205 in the ORF2 library, and 221 in the ORF3 library. The four libraries had 184 common miRNAs, while 2 (ssc-miR-194b-5p, −7143-3p), 4 (ssc-miR-106a, −1277, −129a, −18b), 5 (ssc-miR-132, −491, −7143-5p, −758, −95) and 6 (ssc-miR-144, −187, −199b-5p, −451, −7137-3p, −874) miRNAs were unique to the control, ORF1, ORF2, and ORF3 libraries, respectively.

The total number (319) of distinct, mature porcine miRNA entries in miRBase was much smaller than those for other mammals, such as humans (2555) and mice (1890). Given the strong conservation of miRNAs across animal species [47], we sought to identify the pig orthologs of human miRNAs by aligning the clean reads to human pre-miRNAs and the corresponding mature miRNAs annotated in miRBase. Among known porcine miRNAs found in our libraries, 196 were identical or nearly identical to the registered human miRNAs with 1 or 2-nt mismatches at either the 5′ or 3′ end. More importantly, we could identify 118 additional orthologous miRNAs for which pre-miRNA sequences perfectly mapped to the porcine genome and satisfied established guidelines for miRNA annotation [36] (Figure 2A and Additional file 5). Among these miRNAs, the expression of miR-25-3p was verified by a splinted ligation-based method [43], while other orthologous but unidentified miRNAs were not detectable by this method (Figure 2B). Amongst the orthologous porcine miRNAs identified, 64 were detected in all the libraries, while 2, 6, 6 and 6 were found only in the control, ORF1, ORF2, and ORF3 libraries, respectively. Consequently, combining all the data from our libraries revealed a total of 356 distinct miRNAs, including 314 conserved and 42 non-conserved miRNAs between humans and pigs (Figure 2A and Additional file 6).

Most of the abundant porcine miRNA sequences identified in this study perfectly matched those registered in the miRBase. However, as observed in our earlier studies [41,42], miRNAs showed variations in sequence length at the 5′ and/or 3′ ends (Additional file 7). These isoforms of individual miRNAs are referred to as isomiRs [33] and showed a wide range of expression levels in each library. The end heterogeneity of the identified isomiRs was more frequently found only at the 3′ end (25.74%) relative to only the 5′ end (3.29%) and both ends (5.87%). This suggests that the 5′ end of a miRNA is much more important than the 3′ end, which is consistent with a crucial role for the seed region in miRNA-mRNA interactions [20]. miRNAs often have end variants differing in sequence length [48]. These isomiRs may arise from imprecise processing or terminal trimming of miRNAs. It is also possible that isomiRs may originate from differential processing of miRNAs encoded by paralogous loci. In either case, the proportion of each isomiR is presumed to be cell or tissue specific and developmentally regulated.

### Analysis and validation of differential miRNA expression

**Figure 3:**
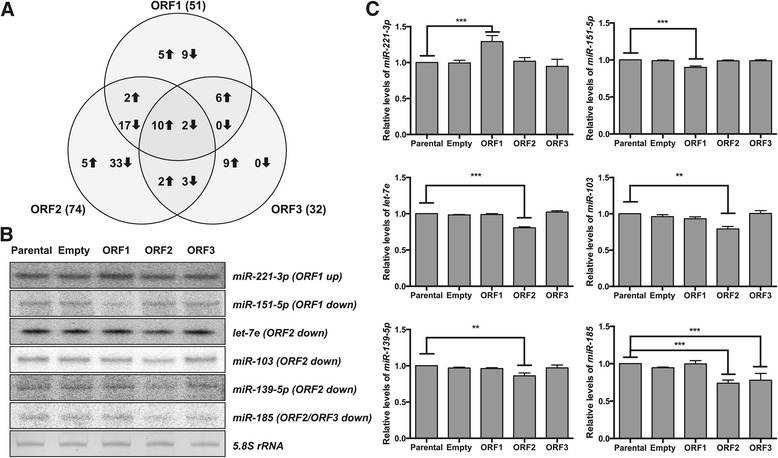
**Effects of PCV2 ORF expression on porcine mature miRNA levels in PK15 cells. A**. Venn diagram showing overlap of PCV2 ORF-regulated miRNAs. Upward and downward arrows indicate up-regulation and down-regulation of miRNAs, respectively. Values listed in parentheses indicate the total number of miRNAs differentially regulated by each ORF, compared to parental PK15 cells. **B**. Splinted ligation analysis of the steady-state levels of mature miRNAs indicated. A representative image from three independent experiments is shown, and parental and empty vector-harboring cells served as controls. Up- or down-regulation of each miRNA by the corresponding ORF(s) is indicated in parentheses. The 5.8S rRNA served as a loading control. **C**. Quantification of miRNA expression levels from the splinted ligation analysis depicted in B. The levels of miRNAs in parental cells were set to 1, and all error bars represent the mean ± SEM from three independent experiments. **P < 0.01, ***P < 0.001 by one-way ANOVA with the Dunnett’s multiple-comparison test.

To identify cellular miRNAs which are differentially expressed between the control and ORF-expressing PK15 cells, miRNA read counts were analyzed for differential expression. A total of 51 miRNAs (23 up-regulated and 28 down-regulated) in the ORF1 library, 74 miRNAs (19 up-regulated and 55 down-regulated) in the ORF2 library, and 32 miRNAs (27 up-regulated and 5 down-regulated) in the ORF3 library exhibited differential expression when compared with the control (Figure 3A and Additional file 8). Remarkably, 12 miRNAs (10 up-regulated and 2 down-regulated) were differentially expressed in all the ORF libraries compared with the control, suggesting a common role in PCV2 replication or pathogenesis. While the largest increase in abundance was observed with let-7c in both ORF1 and ORF3 libraries, and with miR-411-5p in the ORF2 library, miR-196a, miR-361-3p, and miR-1224-5p showed the largest decrease in abundance in the ORF1, ORF2 and ORF3 libraries, respectively. Taken together, our results suggest that upon PCV2 infection of host cells, each ORF can differentially regulate distinct sets of cellular miRNAs, as well as common miRNA subsets.

We next verified the altered expression of ORF-regulated miRNAs by splinted ligation assay [43]. Seven miRNAs, miR-25-3p (down-regulated by ORF1 and ORF2), miR-221-3p (up-regulated by ORF1), miR-151-5p (down-regulated by ORF1), let-7e, miR-103, miR-139-5p (all three down-regulated by ORF2), and miR-185 (down-regulated by ORF2 and ORF3), were randomly selected for verification. Although the extent of changes in expression level as assessed by splinted ligation assay was not identical to the sequencing results, all miRNAs tested showed significantly differential expression patterns (Figures 2B, 3B and C).

miRNAs are often clustered in the genome and can be generated by processing of a common polycistronic transcript [47,49]. Thus, we grouped pre-miRNAs representing mature miRNAs identified in this study into clusters based on genomic location, where miRNAs in each cluster were <10 kb apart. A total of 109 miRNAs were organized into 32 clusters, which were distributed on 14 different chromosomes (Additional file 9). The relative levels of individual mature miRNAs present in these clusters were then analyzed for their expression patterns in control and ORF-expressing PK15 cells. Among the miRNA clusters identified, the *miR-99a-let-7c* cluster exhibited a similar pattern of miRNA expression in response to the PCV2 ORFs. Both miR-99a and let-7c were up-regulated by PCV2 ORF1 and ORF3, suggesting that these miRNAs are functionally related. In contrast, miRNAs belonging to the other clusters showed variable expression patterns. As exemplified by the *miR-99b-let-7e-miR-125a* cluster, not all miRNAs located in a cluster were differentially regulated by the corresponding ORF in the same direction. These results are consistent with independent regulation of post-transcriptional processing of individual miRNAs within a cluster [50]. Overall, our data suggest that the majority of miRNAs in host cells is regulated by PCV2 ORFs at both transcriptional and post-transcriptional levels.

### Prediction and functional analysis of target genes for PCV2 ORF-regulated miRNAs

To understand the biological roles of PCV2 ORF-regulated miRNAs, we predicted their target genes using miRecords, a program that integrates various miRNA target prediction tools [38]. Because the current version of miRecords does not include porcine genes, miRNA target genes were predicted using the human database, assuming that the miRNA-binding sites in the 3′ UTRs of orthologous mRNAs are conserved between humans and pigs. Indeed, it was previously reported that most mammalian mRNAs contain conserved miRNA target sites [25]. We only considered genes that were predicted by at least five of the eleven established miRNA target prediction tools integrated into miRecords [38], since these were the most probable targets of differentially expressed miRNAs. Based on these stringent criteria, 1816 target genes were predicted for miRNAs up-regulated by ORF1, 1286 for miRNAs up-regulated by ORF2, and 1930 for miRNAs up-regulated by ORF3; whereas, 1245, 2261, and 91 genes were identified as putative targets for miRNAs down-regulated by ORF1, ORF2, and ORF3, respectively (Additional file 10).

**Figure 4:**
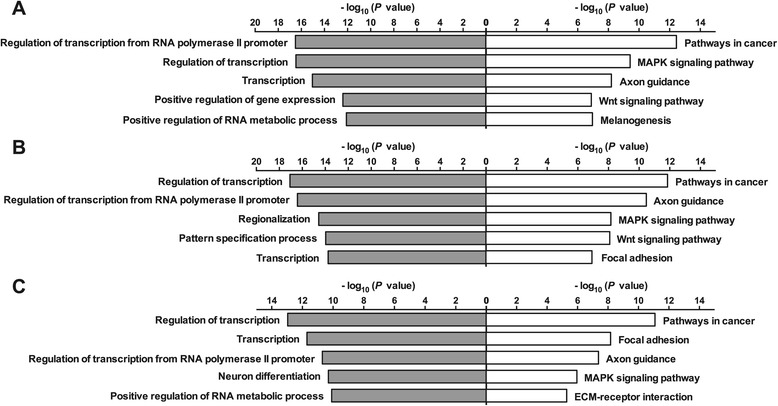
**Functional enrichment analyses on the predicted targets of PCV2 ORF-regulated miRNAs.** The top five most significant GO biological processes (left) and KEGG pathways (right) enriched in the putative target genes of miRNAs regulated by ORF1 **(A)**, ORF2 **(B)**, and ORF3 **(C)** were sorted by the negative log_10_ of the *P* value.

To identify the relevant biological processes and pathways associated with ORF-regulated miRNAs, we performed GO and KEGG pathway analyses with datasets comprising all the putative targets of individual miRNAs regulated by each ORF. The five most enriched categories in individual datasets from each analysis are listed in Figure 4. Intriguingly, transcriptional regulation [GO IDs: 0006355 (regulation of transcription), 0006357 (regulation of transcription from RNA polymerase II promoter), and 0010628 (transcription)] was identified as the most significantly enriched biological process across all the datasets. Furthermore, pathways with altered regulation in cancers [KEGG ID: hsa05200], mitogen-activated protein kinase (MAPK) signaling pathways [KEGG ID: hsa04010], and pathways involved in axon guidance [KEGG ID: hsa04360] were also enriched in all the datasets. Of note, previous work shows that MAPK signaling pathways are activated in PCV2-infected PK15 cells [51,52]. Additional GO and KEGG pathway analyses that were performed separately with the putative targets of miRNAs either up-regulated or down-regulated by each ORF led to similar results. The only exception was with ORF3-down-regulated miRNA targets due to their relatively small number (Additional file 11). These results suggest that deregulation of a host cell’s transcription and signaling pathways through alterations in cellular miRNA levels might be important for PCV2 infection.

### Regulation of ZNF265 and RGS16 by PCV2 ORF2-regulated miR-139-5p and let-7e

**Figure 5:**
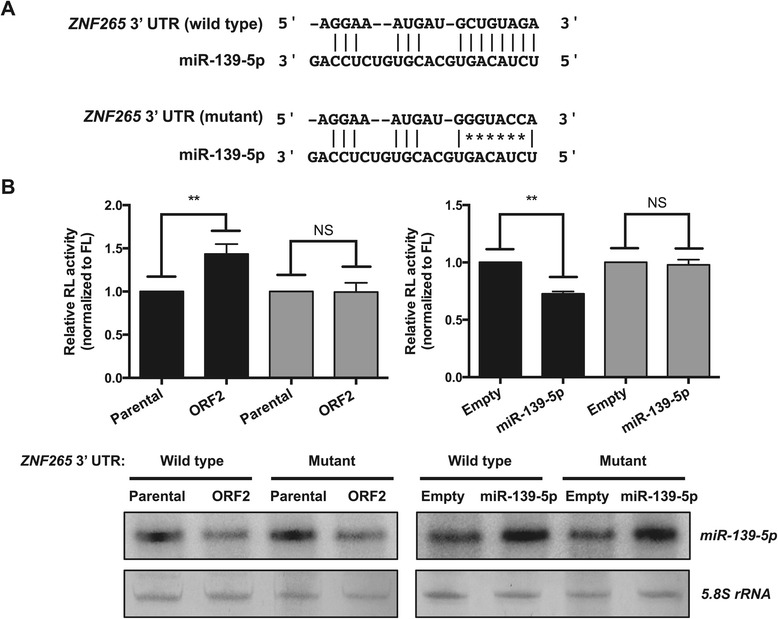
**Regulation of** ***ZNF265*** **by miR-139-5p. A**. Potential wild-type miR-139-5p target site (top) and a mutant with seed mismatches indicated by asterisks (bottom) in the *ZNF265* 3′ UTR. **B**. Relative luciferase activity from the *Renilla*-luciferase reporter constructs containing either the wild-type (black bars) or mutated (gray bars) *ZNF265* 3′ UTR in parental and ORF2-expressing PK15 cells (top left), and in parental PK15 cells with or without (empty vector) miR-139-5p overexpression (top right). The level of mature miR-139-5p in each sample was also determined by splinted ligation assay (bottom). The 5.8S rRNA served as a loading control. Note that miR-139-5p is down-regulated in ORF2-expressing cells compared with parental cells (bottom left). For quantification, *Renilla*-luciferase activity was normalized to firefly-luciferase activity. The normalized activity of *Renilla* luciferase in parental cells or the same cells transfected with the empty vector was set to 1. Data are presented as the mean ± SEM from three independent experiments. NS, no significance; **P < 0.01 by Student’s *t*-test. Abbreviations: RL, *Renilla* luciferase; FL, firefly luciferase.

Among the putative targets of the ORF-regulated miRNAs, *zinc finger protein 265* (*ZNF265*) and *regulator of G protein signaling 16* (*RGS16*) were of particular interest because proteins encoded by the porcine orthologs of these genes have been reported to interact with PCV2 ORF1 (Rep) and ORF3 proteins, respectively [13,53]. The porcine *ZNF265* 3′ UTR contains a putative target site for miR-139-5p, whereas the porcine *RGS16* 3′ UTR was predicted to have a single site targeted by the let-7 miRNA family members let-7b-5p, let-7c and let-7e (Table 2). These predicted sites were capable of stably pairing both at the 3′ end of the corresponding miRNAs as well as to the seed region. First, we tested potential regulation of *ZNF265* by miR-139-5p using a *Renilla*-luciferase reporter linked to the wild-type or mutated *ZNF265* 3′ UTR (Figure 5A). Down-regulation of miR-139-5p in ORF2-expressing PK15 cells led to ~43% increase in *Renilla*-luciferase activity from the wild-type *ZNF265* 3′ UTR reporter, compared with parental cells (*P* < 0.01; Figure 5B, left panel, black bars). Conversely, overexpression of miR-139-5p in parental PK15 cells reduced the *Renilla*-luciferase activity by ~27%, relative to cells transfected with the empty vector (*P* < 0.01; Figure 5B, right panel, black bars). None of these effects were seen with a mutated *ZNF265* 3′ UTR reporter in which the miR-139-5p target site had seed mismatches (Figures 5B, gray bars). These results indicate that *ZNF265* is a target regulated by miR-139-5p through its 3′ UTR.

**Table 2:** **Potential miRNA target sites within the 3**′ **UTR of** ***ZNF265*** **and** ***R*** ***GS16*** **mRNA**

**Putative target gene**	**miRNA**	**miRNA-mRNA interaction**	**Δ** ***G*** **(kcal/mol)**
*ZNF265*	miR-139-5p	target 5′ AGGAA––AUGAU–GCUGUAGAC 3′	−20.20
		| | | | | | | | | | | | | |	
		miRNA 3′ GACCUCUGUGCACGUGACAUCU 5′	
*RGS16*	let-7b-5p	target 5′ GAGCUGGCAGCCUGACUGGCUCC 3′	−28.00
		| | | || | | | | | | | | | | | |	
		miRNA 3′ UUGGUGUGUUGGA–UGAUGGAGU 5′	
	let-7c	target 5′ GAGCUG–GCAGCCUGACUGGCUCC 3′	−24.20
		| | | | | | | | | | | | | | | | | | |	
		miRNA 3′ UUGGUAUGUUGGA–UGAUGGAGU 5′	
	let-7e	target 5′ GAGCUG–GCAGCCUGACUGGCUCC 3′	−24.20
		| | | | | | | | | | | | | | | | | |	
		miRNA 3′ UUGAUAUGUUGGAG–GAUGGAGU 5′

**Figure 6:**
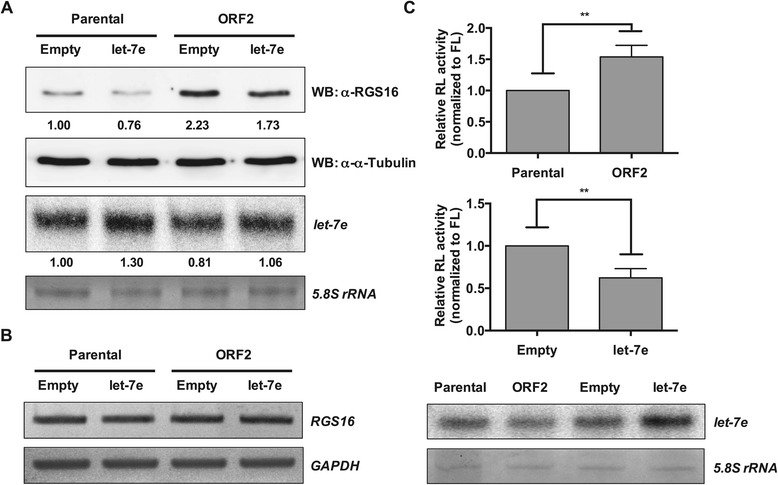
**let-7e regulates** ***RGS16*** **. A**. Western blot (WB) analysis of RGS16 in parental and ORF2-expressing PK15 cells in the absence (empty vector) or presence of let-7e overexpression. α-Tubulin served as an internal control. Mature let-7e levels were also measured by splinted ligation assay. The 5.8S rRNA served as a loading control. Note that let-7e is down-regulated in ORF2-expressing cells (lane 3) relative to parental cells (lane 1). Values below the images indicate quantified signal intensities, and the level of RGS16 protein or let-7e in parental cells transfected with the empty vector was set to 1. **B**. Semi-quantitative RT-PCR analysis of *RGS16* and *GAPDH* mRNA levels in parental and ORF2-expressing cells in the absence (empty vector) or presence of let-7e overexpression. No significant difference in *RGS16* mRNA levels was detected between samples. *GAPDH* was used as an internal control. **C**. Relative luciferase activity from the *Renilla* luciferase-*RGS16* 3′ UTR reporter in parental and ORF2-expressing PK15 cells (top), and in parental PK15 cells in the absence (empty vector) or presence of let-7e overexpression (middle). The level of mature let-7e in each sample was also determined by splinted ligation assay (bottom). The 5.8S rRNA was used as a loading control. *Renilla*-luciferase activity was normalized to firefly-luciferase activity, and the normalized activity of *Renilla* luciferase in parental cells (top) or the same cells transfected with the empty vector (middle) was set to 1. All error bars indicate the mean ± SEM from three independent experiments. **P < 0.01 by the Student’s *t*-test. Abbreviations: RL, *Renilla* luciferase; FL, firefly luciferase.

*RGS16* was predicted to be a target of let-7b-5p and let-7e, both down-regulated by ORF2, and let-7c, which was up-regulated by both ORF1 and ORF3 (Table 2 and Additional file 8). Thus, we next assessed the potential regulation of *RGS16* through the miRNA target site within its 3′ UTR by focusing on let-7e, because the abudance of let-7c was extremly low, and let-7b-5p was less abundant than let-7e with similar expression changes (Additional file 8). Western blot analysis revealed that down-regulation of let-7e in ORF2-expressing PK15 cells increased the level of RGS16 by more than two-fold compared with parental cells (Figure 6A; compare lanes 1 and 3). In contrast, let-7e overexpression reduced the level of RGS16 by ~22-24% in both parental and ORF2-expressing cells (Figure 6A; compare lanes 2 and 4). However, in all cases, the steady-state levels of *RGS16* mRNA transcripts were unchanged as determined by semi-quantitative RT-PCR (Figure 6B). Collectively, the inverse correlation of RGS16 expression at the protein level with let-7e expression levels suggested that *RGS16* could be targeted by let-7e for repression. To test this possibility, we performed luciferase assays with a reporter construct expressing *Renilla*-luciferase mRNA bearing the *RGS16* 3′ UTR. ORF2-expressing PK15 cells, which had reduced let-7e levels, showed an approximately 54% increase in *Renilla*-luciferase activity, compared to parental cells (*P* < 0.01; Figure 6C, top panel). In contrast, let-7e overexpression in parental PK15 cells caused ~38% reduction of *Renilla*-luciferase activity, relative to cells transfected with the empty vector (*P* < 0.01; Figure 6C, middle panel). Taken together, these results suggest that *RGS16* is a target of let-7e-dependent post-transcriptional repression via its 3′ UTR.

## Discussion

PCV2 is linked to PMWS and other porcine diseases, which greatly affect the global pig industry. Cellular miRNAs have been demonstrated to be key regulators of virus-host interactions, and their expression is often deregulated by viral infections [27-29]. However, it has remained unknown whether this occurs during PCV2 infection. In this study, we used deep sequencing technology to analyze cellular miRNAs with significantly altered abundance as a consequence of expressing three individual PCV2-encoded ORF proteins in PK15 cells. Distinct subsets of cellular miRNAs were either positively or negatively regulated by each ORF. Some of these miRNAs may have antiviral activity, while others function to reshape the cellular environment to benefit viral replication. Individual PCV2 ORF proteins could affect different steps of miRNA biogenesis through a direct or indirect mechanism at both transcriptional and post-transcriptional levels. First, they may influence miRNA expression at the transcriptional level. The PCV2 ORF1-encoded Rep protein has been known to interact with two distinct cellular proteins linked to transcriptional regulation [12,13]: c-Myc, a transcriptional regulator, and a DNA repair protein, thymine DNA glycosylase which associates with transcriptional activators and coactivators such as CBP/p300, thyroid transcription factor-1, and estrogen receptor-alpha [54-56]. Therefore, such interactions of PCV2-encoded proteins with cellular proteins that play a role in transcriptional regulation are speculated to modulate the expression of a subset of miRNAs by either inhibiting or promoting the activity of the transcriptional regulators. Similarly, unidentified RNA binding proteins that interact with PCV2-encoded proteins may mediate specific regulation of post-transcriptional miRNA processing. This hypothesis is supported by the observation that a number of RNA binding proteins are known to modulate processing of pri- and/or pre-miRNA intermediates into mature miRNAs in a context-dependent manner [57,58]. Given that the opposing activities of miRNA biogenesis and degradation determine the steady-state levels of miRNAs, interactions between PCV2-encoded proteins and cellular proteins can also affect miRNA stability. Combinatorial effects of all these events might contribute to differential expression of cellular miRNAs in the presence of individual PCV2 ORF proteins.

Viruses can subvert host cell functions at several levels, including an alteration in transcription patterns of cellular genes. Indeed, the GO analysis of the potential targets of ORF-regulated miRNAs identified transcriptional regulation as the most significantly enriched biological process. This suggests that a set of cellular miRNAs might be coordinately regulated during PCV2 infection to have a profound effect on transcription of host cells. Since miRNAs act as fine-tuners to maintain the optimal level of gene expression [58], it is likely that PCV2 ORF proteins control gene expression of host cells by perturbing a network of cellular miRNAs that form multiple layers of positive- and negative-feedback circuits with transcriptional regulators. These ultimate changes in transcriptional regulation may partially account for the extensive changes in cellular gene expression previously observed during PCV2 infection [59-61]. During infection, many viruses exert control over a variety of host signaling pathways to support their successful replication [62]. Although most cellular responses to viral infection are initiated as defense mechanisms, the virus could eventually exploit a subset of these activities to ensure efficient replication. Not surprisingly, the KEGG pathway analysis revealed a significant enrichment for MAPK signaling in the putative targets of PCV2 ORF-regulated miRNAs. Viruses often target the MAPK signaling pathways, which are critical for many cellular processes [51,52,63-65]. PCV2 specifically activates the c-Jun NH_2_-terminal kinases (JNK1/2) and p38 in infected PK15 cells, and inhibition of these MAPK pathways significantly hindered viral transcription, viral protein synthesis, viral progeny release, and virus-induced apoptosis [51]. The extracellular signal-regulated kinase (ERK) signaling pathway, which is one of the MAPK cascades, was also found to be activated in PCV2-infected PK15 cells, and its inhibition led to a reduction of viral gene expression and progeny release [52]. Intriguingly, the ERK pathway enhances miRNA production by phosphorylating trans-activation response RNA-binding protein (TRBP), a well-characterized interacting partner of Dicer, which stabilizes the Dicer-TRBP complex [66]. In mammals, several cellular miRNAs mediate the coordinated repression of genes in a shared pathway [67]. Therefore, several miRNAs whose abundance is affected by PCV2 ORFs may converge to coordinate regulation of components in the MAPK signaling pathways, leading to changes in signal output that could be beneficial for PCV2 infection.

In this study, several lines of evidence demonstrate that porcine *ZNF265* and *RGS16* are targets of miR-139-5p and let-7e, respectively, both of which are down-regulated by ORF2. ZNF265 and RGS16 proteins were found to interact with PCV2 ORF1-encoded Rep and ORF3 proteins, respectively [13,53]. ZNF265 is a spliceosomal protein that associates with mRNA and splicing factors [68]. Hence, interaction of the ORF1 protein Rep with ZNF265 was previously suggested to affect transcription and splicing in host cells [13]. RGS proteins attenuate signaling via G-protein coupled receptors associated with the regulation of numerous cellular processes by accelerating the intrinsic GTPase activity of heterotrimeric G proteins [69]. RGS16, a member of the RGS protein subfamily, was proposed to be involved in the nuclear translocation of the PCV2 ORF3 protein [53]. Based on our results, ORF2-induced down-regulation of miR-139-5p and let-7e is likely to augment the expression of *ZNF265* and *RGS16* during PCV2 infection. Consequently, the changes in gene expression could affect a wide variety of cellular processes associated with ZNF265 and RGS16, as well as the interaction with their respective ORF protein, thereby influencing a host cell’s response to PCV2 infection.

Porcine monocyte/macrophage lineage cells are the primary targets of PCV2 in vivo [70]. Hence, it would be interesting to investigate whether PCV2-encoded proteins can alter cellular miRNA profiles in porcine macrophages as we have observed in PK15 cells. If so, a comparative analysis between PK15 cells and porcine macrophages will reveal the differences and similarities in PCV2 ORF-regulated cellular miRNAs between these cell types, which provides a resource to further delineate the potential role of the ORF-regulated miRNAs in PCV2 replication and pathogenesis.

In conclusion, we identified cellular miRNAs that are differentially regulated by the three major PCV2 ORF proteins, although the underlying mechanisms and functional relevance of these miRNAs in virus-host interactions remain to be determined. The putative targets of the ORF-regulated miRNAs were mainly associated with transcriptional regulation and signaling pathways with altered regulation in distinct aspects of the viral life cycle as well as in cancers. Furthermore, we validated *ZNF265* and *RGS16*, whose proteins interact with PCV2-encoded proteins, as target genes of miR-139-5p and let-7e, respectively, which are both down-regulated by ORF2. Taken together, our results suggest that miRNA-mediated regulation of gene expression may play a crucial role in modulating the activity of host cells for PCV2 replication and pathogenesis.

## Additional files

Additional file 1:
**Sequences of primers used in this study.** List of primers used for generation of PK15 stable cell lines expressing each of the three PCV2 ORFs, verification of ORF expression by RT-PCR, and construction of plasmids for miRNA overexpression and luciferase reporter assays. Additional file 2:
**Sequences of bridge oligonucleotides used for splinted ligation assay.** List of bridge oligonucleotides used for splinted ligation assay by which differential expression of ORF-regulated miRNAs was validated. Additional file 3:
**Generation of PK15 cell lines stably expressing each PCV2 ORF.** A. Construction of recombinant retroviral vectors expressing each ORF protein under the control of the CMV promoter. Shading highlights the ORF3-coding region embedded within the ORF1-coding region in the antisense orientation. The annealing sites for the primers used for PCR following RT are indicated. The illustrations are not drawn to scale. B. Agarose gel electrophoresis showing expression of each ORF-coding gene in the respective stable PK15 cell line. RT-PCR analysis was performed to detect *ORF* mRNA transcripts. Lane 1, parental cells; Lane 2, cells harboring empty vector; Lane 3, ORF1-expressing cells; Lane 4, ORF2-expressing cells; Lane 5, ORF3-expressing cells. Parental and empty vector-harboring cells served as controls, and *GAPDH* was used as an internal control. RT was performed with total RNA from each sample using random primers, and the resulting cDNAs were amplified by PCR. As indicated in A, the ORF1 cDNA was PCR amplified using the ORF1/3-RT-F and ORF1-RT-R primers (473 bp amplicon) or ORF1/3-RT-F and ORF1/3-RT-R primers (174 bp amplicon), the ORF2 cDNA using the ORF2-RT-F and ORF2-RT-R primers (342 bp amplicon), and the ORF3 cDNA using the ORF1/3-RT-F and ORF1/3-RT-R primers (174 bp amplicon). Note that the ORF1/3-RT-F and ORF1/3-RT-R primers anneal to identical but oppositely oriented sites on the complementary strands of ORF1 and ORF3 cDNAs, giving rise to RT-PCR products with the same size. Additional file 4:
**Relative abundances of known porcine miRNAs in each library.** List of read counts for 238 known porcine miRNAs in each library. Additional file 5:
**List of miRNAs orthologous between humans and pigs that were identified in this study.** Sequence information for the 118 orthologous porcine miRNAs identified in this study but unannotated in miRBase along with their corresponding precursors and genomic locations. Additional file 6:
**Relative abundances of all the identified porcine miRNAs in each library.** List of read counts for 356 known and orthologous porcine miRNAs in each library. Additional file 7:
**Alignment of mature miRNA sequences for identifying isomiRs.** Sequence information and read counts for isomiRs that differ in sequence length at the 5′ and/or 3′ ends in each library. Additional file 8:
**Differential regulation of porcine miRNAs by individual PCV2 ORF proteins.** Fold change and statistical analyses of miRNA read counts were performed to identify ORF-regulated miRNAs. Additional file 9:
**Expression patterns of clustered miRNAs.** Expression patterns of individual miRNAs in 32 clusters identified in this study. Additional file 10:
**Predicted targets of miRNAs up- or down-regulated by PCV2 ORFs.** List of target genes of ORF-up-regulated or ORF-down-regulated miRNAs that were predicted by at least five of the eleven established miRNA target prediction tools integrated into miRecords. Additional file 11:
**The top-10 GO biological processes and KEGG pathways enriched in the putative targets of PCV2 ORF-regulated miRNAs.** List of the ten most enriched categories of GO annotations and KEGG pathways corresponding to the putative targets of ORF-up-regulated or ORF-down-regulated miRNAs.
