# Supplementary figures and images for: Changes in cellular microRNA expression induced by porcine circovirus type 2-encoded proteins
Source: Vet Res. 2015 Apr 10;46(1):39. doi: 10.1186/s13567-015-0172-5 (PMC4391141; doi:10.1186/s13567-015-0172-5)

**A**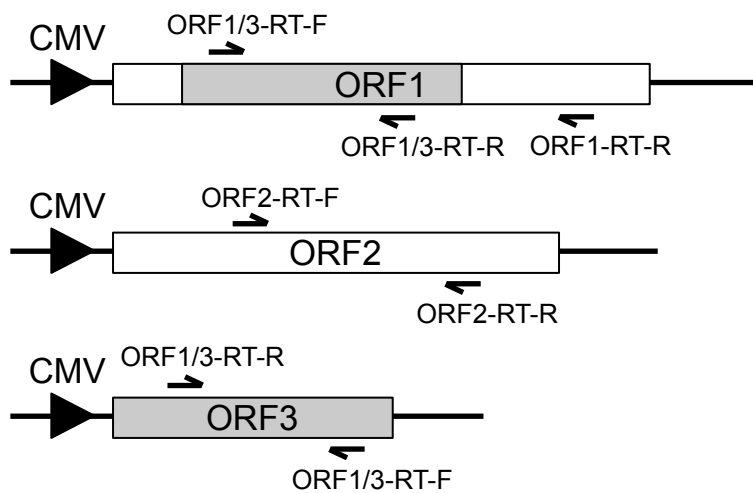**B**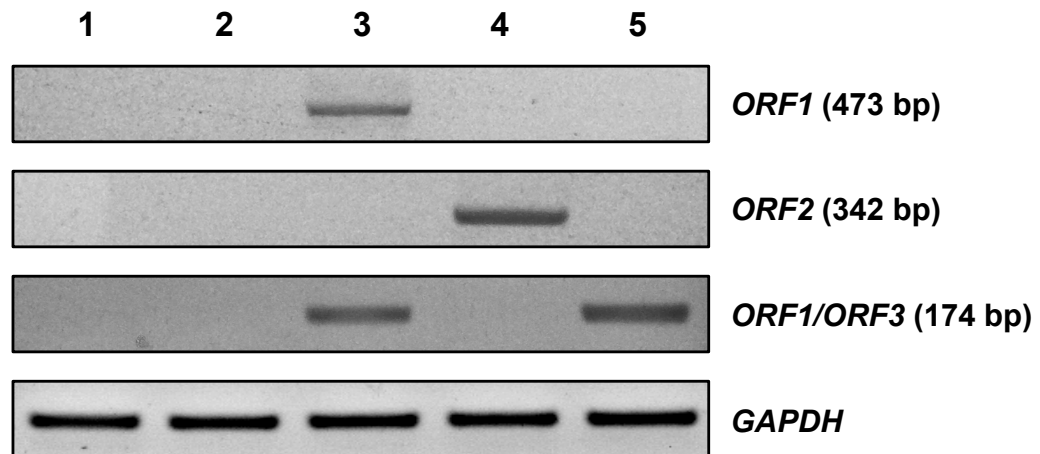

Supplement: Additional file 3: — Generation of PK15 cell lines stably expressing each PCV2 ORF. A. Construction of recombinant retroviral vectors expressing each ORF protein under the control of the CMV promoter. Shading highlights the ORF3-coding region embedded within the ORF1-coding region in the antisense orientation. The annealing sites for the primers used for PCR following RT are indicated. The illustrations are not drawn to scale. B. Agarose gel electrophoresis showing expression of each ORF-coding gene in the respective stable PK15 cell line. RT-PCR analysis was performed to detect ORF mRNA transcripts. Lane 1, parental cells; Lane 2, cells harboring empty vector; Lane 3, ORF1-expressing cells; Lane 4, ORF2-expressing cells; Lane 5, ORF3-expressing cells. Parental and empty vector-harboring cells served as controls, and GAPDH was used as an internal control. RT was performed with total RNA from each sample using random primers, and the resulting cDNAs were amplified by PCR. As indicated in A, the ORF1 cDNA was PCR amplified using the ORF1/3-RT-F and ORF1-RT-R primers (473 bp amplicon) or ORF1/3-RT-F and ORF1/3-RT-R primers (174 bp amplicon), the ORF2 cDNA using the ORF2-RT-F and ORF2-RT-R primers (342 bp amplicon), and the ORF3 cDNA using the ORF1/3-RT-F and ORF1/3-RT-R primers (174 bp amplicon). Note that the ORF1/3-RT-F and ORF1/3-RT-R primers anneal to identical but oppositely oriented sites on the complementary strands of ORF1 and ORF3 cDNAs, giving rise to RT-PCR products with the same size. [file 13567_2015_172_MOESM3_ESM.pdf]
